# The role of mTOR in age-related diseases

**DOI:** 10.1080/14756366.2021.1955873

**Published:** 2021-07-26

**Authors:** Zofia Chrienova, Eugenie Nepovimova, Kamil Kuca

**Affiliations:** Department of Chemistry, Faculty of Science, University of Hradec Kralove, Hradec Kralove, Czechia

**Keywords:** Ageing, mTORC1, mTORC2, age-related disease, rapamycin

## Abstract

The ageing population is becoming a significant socio-economic issue. To address the expanding health gap, it is important to deepen our understanding of the mechanisms underlying ageing in various organisms at the single-cell level. The discovery of the antifungal, immunosuppressive, and anticancer drug rapamycin, which possesses the ability to extend the lifespan of several species, has prompted extensive research in the areas of cell metabolic regulation, development, and senescence. At the centre of this research is the mTOR pathway, with key roles in cell growth, proteosynthesis, ribosomal biogenesis, transcriptional regulation, glucose and lipid metabolism, and autophagy. Recently, it has become obvious that mTOR dysregulation is involved in several age-related diseases, such as cancer, neurodegenerative diseases, and type 2 diabetes mellitus. Additionally, mTOR hyperactivation affects the process of ageing per se. In this review, we provide an overview of recent insights into the mTOR signalling pathway, including its regulation and its influence on various hallmarks of ageing at the cellular level.

## 1. Introduction

**Figure 1.**
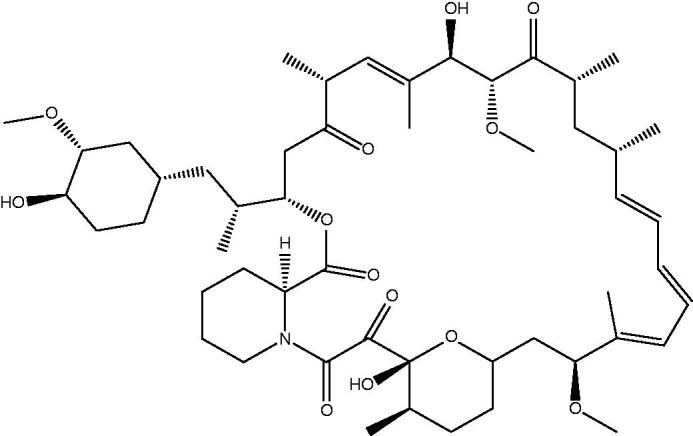
Chemical structure of rapamycin.

Collecting soil samples on Easter Island (Rapa Nui) in the 1970s, no one could predict that bacterial cultures of *Streptomyces hygroscopicus* isolated from these samples would change the way we look at the cell cycle and help us better understand several signalling pathways and cellular processes. The isolated strain demonstrated inhibitory effects on *Candida albicans, Microsporum gypseum, Trichophyton granulosum,* as well as other gram-positive bacteria, while all gram-negative species were resistant. It was soon shown that *S. hygroscopicus* produces an antibiotic with antifungal properties, named rapamycin after its place of discovery (Figure 1). This substance, also known by its generic name sirolimus, is a lipophilic macrolide with a white crystalline structure that is insoluble in water but readily soluble in organic solvents^1–3^.

Rapamycin also shows antiproliferative effects in a wide range of taxa, from yeast to mammals. Moreover, Dr. Sehgal of the Ayerst Research Laboratories in Montreal (where the strain was first isolated) observed that the compound has anti-tumour activity, opening a new window for anticancer drug development^4^.

The immunosuppressive activity of rapamycin was first evidenced by its ability to decrease humoral IgE production in rats and its preventive effects in two animal models of human autoimmune diseases^5^. Only the discovery of the immunosuppressive drug FK506 (tacrolimus) by the Fujisawa Pharmaceutical Laboratories drew more attention than rapamycin as an immunosuppressant. Both FK506 and rapamycin contain macrolactam rings with hemiketal-masked diketopipecolic acid amidic components that bind to the identical family of intracellular receptors. Despite these similarities, the mechanism of action of rapamycin is distinct from that of FK506. While FK506 binds to the immunophilin (peptidyl-prolyl *cis/trans* isomerase FKBP12, E.C. 5.2.1.8) receptor resulting in an immunophilin–drug complex with inhibitory effects against the activity of calcineurin (T cell antigen receptor activating enzyme), the rapamycin–immunophilin complex interferes with T cell growth factors. Nevertheless, both rapamycin and tacrolimus have significant clinical implications, since both are approved for use in prophylaxis for renal rejection^6–9^.

The exceptional antifungal, anticancer, and immunosuppressant properties of rapamycin have also been linked to its capacity to delay cell cycle progression at the G1 phase^10^. These effects were later related to the inhibition of large multiprotein complexes with serine/threonine kinase activity, called target of rapamycin (TOR)^11^. In 2003, Vellai and colleagues revealed for the first time the role of TOR signalling in ageing. The downregulation of TOR signalling extends lifespan in *Saccharomyces cerevisiae*, *Caenorhabditis elegans*, and *Drosophila melanogaster*^12–14^. In 2009, a study showed that the treatment of genetically heterogenous male and female mice with rapamycin extends mean and maximum lifespans, despite the initiation of treatment at an advanced age (corresponding to a human age of 60 years)^15^. Several additional studies have shown a positive impact of rapamycin on lifespan in different genetically modified mouse strains^16–20^. Accordingly, rapamycin is a promising candidate for a mammalian longevity drug.

In this review, we summarise our current understanding of the roles of the mTOR pathway in ageing. In particular, we describe the structures, regulation, and functions of mTOR complex 1 (mTORC1) and mTOR complex 2 (mTORC2), with a focus on their effects on relevant cellular processes, as well as their associations with various age-related diseases.

## 2. Overview of mTOR complex 1 and mTOR complex 2

Cell proliferation and cell growth are two diverse yet connected processes involved in tissue, organ, and organismal development. Hartwell’s study of yeast mutants in the late 1960s, a clear distinction between these two processes was established^21^. Owing to Hartwell’s intense focus on cell division, our understanding of the central regulator of cell proliferation—cyclin-dependent kinases (CDK)—is quite extensive. However, cell growth and its regulation were largely neglected until the late 1990s. Cell growth is not a mere accumulation of cell mass controlled by nutrients. Rather, it is a complex process of balanced macromolecular synthesis with a crucial role in cell physiology controlled by signalling pathways, among which TOR kinase plays a central role^22^.

Originally, TOR was identified from yeast mutants. However, it is highly conserved from yeast to mammals. In yeast, it is encoded by two genes, *TOR1* and *TOR2*^23^, while the human genome contains a single *mTOR* gene mapped to chromosomal band 1p36.2^24^.

The mechanistic (formerly mammalian) target of rapamycin (mTOR), also known as RAFT, FRAP, or RAPT, was subsequently discovered by three independent groups in 1994 and 1995^11^^,^^25–27^. This 289-kDA serine/threonine kinase is a part of the phosphatidylinositol 3-kinase-related kinase (PIKK) family. This family includes several kinases involved in cell growth and cell cycle control, the maintenance of telomere length, DNA damage checkpoints, and recombination^28^. The mTOR protein consists of several domains, including at least 20 HEAT repeats (Huntingtin-Elongation factor 3-regulatory subunit of protein phospahase-2A, and TOR1) in the *N*-terminal region, the FAT domain (FKBP-associated protein/ataxia-telangiectasia mutated/transactivation-transformation domain-associated protein), which binds to Deptor (regulatory protein), the FRB domain (FKBP-rapamycin binding domain), which is the docking site for immunophilin-rapamycin inhibitory complex, kinase domain, and the FATC domain (FRAP/ATM/TRRAP/Carboxy terminal), which is involved in substrate recognition^29^.

The crystal structure of rapamycin-FKBP12 attached to the FRB domain of TOR revealed extensive interactions between the FRB domain and rapamycin, while the interaction between the FRB domain and immunophilin FKBP12 is limited, indicating that rapamycin is presented to TOR in a favourable conformation by FKBP12^30^. The inhibitory effect of rapamycin on mTOR may be a consequence of the allosteric reduction of kinase domain-specific activity or a deficiency in the structural stability of the protein complex.

In eukaryotic cells, two large and functionally diverse multiprotein complexes have been identified: mTORC1 and mTORC2. Not only do the two complexes phosphorylate different substrates, contributing to various physiological functions, but their sensitivity to rapamycin differs. While mTORC1 is rapamycin-sensitive, mTORC2 is insensitive to acute rapamycin therapy^31^. However, chronic exposure to the compound has a negative effect on mTORC2 activity. A recent proteomic analysis identified a novel mTOR complex containing GIT1 (GPCR kinase-interacting protein 1)^32^. Focussing on the relatively well-established complexes, we provide detailed descriptions of the structures, upstream regulators, and downstream effectors of mTORC1 and mTORC2.

### 2.1. mTORC1—structure and function

**Figure 2.**
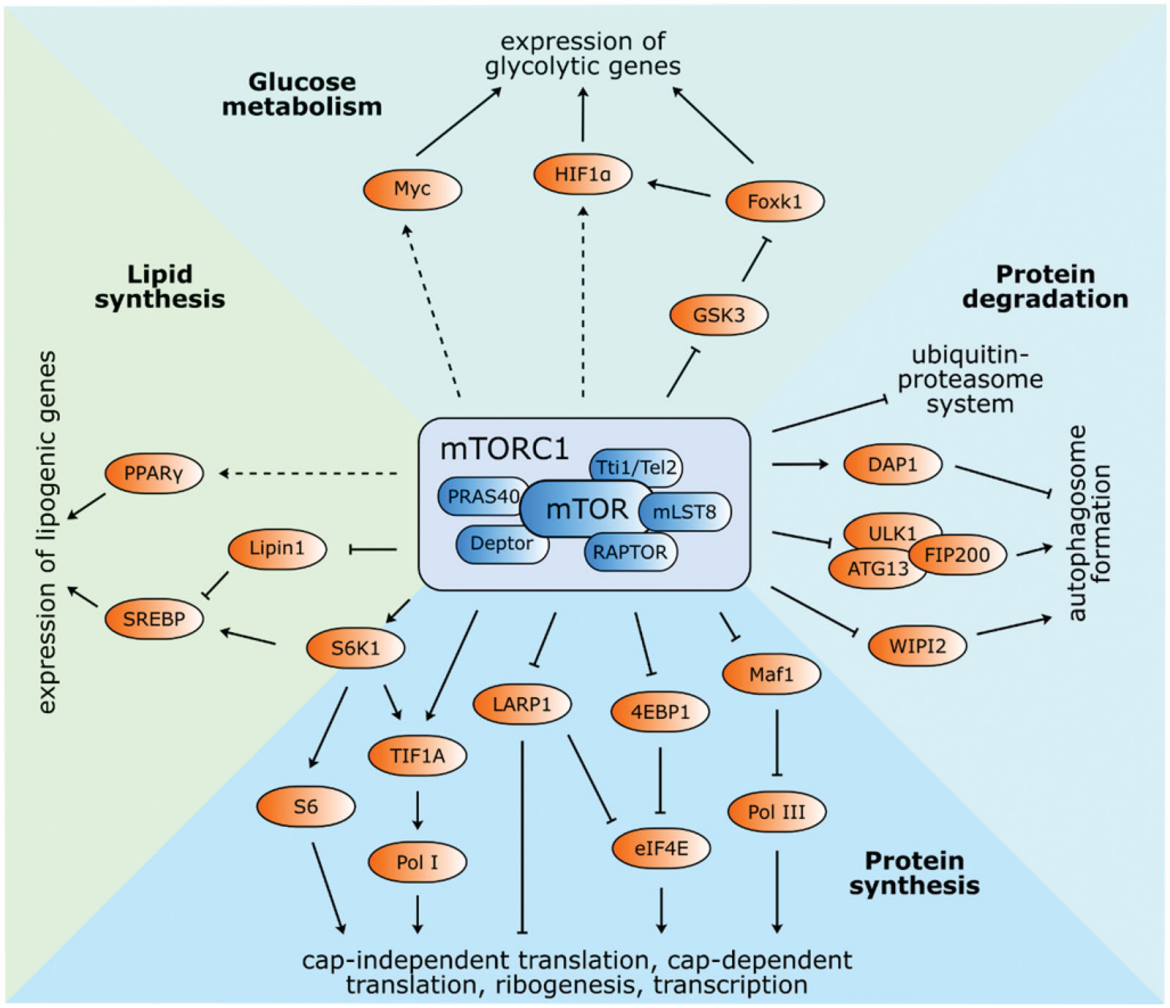
Structure and functions of mTOR complex 1. The best-known targets of mTORC1 phosphorylation S6K1 and 4EBP1 have a vital function in protein synthesis. Phosphorylated S6K1 consequently phosphorylates ribosomal protein S6 and commences cap-independent translation. Activated 4EBP1 relieves its inhibitory function towards eIF4E and initiates cap-dependent translation. Ribosomal biogenesis is enhanced by stimulated translation of 5′TOP mRNA *via* 4EBP1 and LARP1. Pol I and Pol III are activated by phosphorylation of TIF1A and inhibition of Maf1, respectively. Autophagy-related ULK1/ATG13/FIP200 protein complex is inhibited by mTORC1 phosphorylation, as well as WIPI2 (positive regulator of autophagy). On the other hand, autophagy suppressor DAP1 is activated. Protein expression of lipid and cholesterol homeostasis-involved genes is managed by transcription factors SREBP and PPAR-γ. mTORC1-mediated phosphorylation of lipin-1 alleviates SREBP inhibition. Several glycolytic enzyme genes are indirectly modulated by mTORC1 *via* transcription factors HIF1α and Myc. Expression of HIF1α is regulated either at the level of translation or *via* inhibited GSK3/Foxk1 pathway.

mTORC1 multiprotein complex (Figure 2) consists of six protein components: mTOR, mammalian lethal SEC13 protein 8 (mLST8)^33^, regulatory-associated protein of TOR (Raptor)^34^, Tti1/Tel2 complex^35^, Proline-rich Akt substrate 1 40 kDa (PRAS40)^36^, and DEP domain-containing mTOR-interacting protein (Deptor)^37^. mLST8 has a vital role in the phosphorylation of clearly established mTORC1 effectors: ribosomal S6 kinase 1 (S6K1) and the eukaryotic initiation factor 4E binding protein 1 (4EBP1)^33^. Raptor and the Tti1/Tel2 complex are both involved in substrate recognition, regulation, and stabilisation of the mTORC1 assembly. Finally, PRAS40 and Deptor are regulatory proteins that inhibit the kinase activity of mTORC1^37^. This multiprotein complex is an important regulator of cell growth and its effects are mediated by several signalling pathways, described in Figure 2 and the following text.

#### 2.1.1. Protein synthesis

Containing a short sequence important for phosphorylation, called a TOR signalling (TOS) motif, both S6K1 and 4EBP1 bind to Raptor^38^^,^^39^. S6K1 is activated by mTORC1 *via* direct Thr389 phosphorylation, which leads to the phosphorylation of ribosomal S6, concluding in cap-independent translation.

The translation of mRNA containing a cap structure (m^7^GpppN moiety) at the 5′end is initiated by the mTORC1 phosphorylation of 4EBP1 on Thr37/46/70 and Ser65. The phosphorylation of 4EBP1 relieves its inhibitory activity towards eukaryotic initiation factor 4E (eIF4E), which might subsequently recognise the cap structure on mRNAs and commence cap-dependent translation^40^.

#### 2.1.2. Ribosome biogenesis and transcription

An additional mechanism by which the proteosynthesis capacity of cells is enhanced is the promotion of ribosomal biogenesis. Ribosomal proteins and other components of the translational apparatus are encoded by mRNAs carrying the 5′ terminal oligopyrimidine (5′TOP) motif with translational regulatory function^41^. The translation of these 5′TOP-capped mRNAs is enhanced by mTORC1 *via* two mechanisms, the phosphorylation of the previously mentioned effector 4EBP1 and La-related protein 1 (LARP1). Curiously, LARP1 may act as both an enhancer and repressor of 5′TOP mRNA translation. As a repressor, LARP1 prevents eIF4F complex formation by disrupting mRNA binding^42^. On the other hand, when phosphorylated by S6K1, LARP1 binds to the 3′UTR of 5′TOP mRNAs and increases translation^43^.

In addition, the synthesis of rRNAs and tRNAs is positively controlled by mTORC1. Mammalian RNA Polymerase I (Pol I) transcribes precursor rRNA that sequentially forms three out of four mature rRNA species (5.8S, 18S, and 28S), while RNA Polymerase III (Pol III) transcribes the fourth RNA species, 5S rRNA, as well as tRNA. Pol I can be activated by the mTORC1 effector S6K1 or by the mTORC1-mediated phosphorylation of transcription initiation factor IA (TIF1A), which is then translocated to the nucleolus, where it can activate Pol I^44^^,^^45^. mTORC1 also phosphorylates (and consequently inhibits) the repressor of RNA polymerase III transcription MAF1 homolog (MAF1)^46^. Moreover, the direct binding of mTORC1 to the promoter region of Pol I and Pol III increases gene expression^47^.

#### 2.1.3. Autophagy

Autophagy, together with the ubiquitin-proteasome system (UPS), maintains the cytoplasmic protein quality in the cell. This catabolic process involving the sequestration and degradation of intracellular components is controlled by mTORC1 *via* the regulation of the ULK1/ATG13/FIP200 protein complex (unc-51-like kinase 1/autophagy-related gene13/focal adhesion kinase family-interacting protein of 200 kDa), which interacts with Raptor *via* ULK1. The phosphorylation of ULK1 and ATG13 inhibits their activity and suppresses autophagosome formation. Autophagy regulation is limited to mTORC1 since ULK1 does not bind to the mTORC2-specific component Rictor (rapamycin-insensitive companion of mTOR)^48^^,^^49^. mTORC1 also regulates autophagy by the activation of the autophagy suppressor death-associated protein 1 (DAP1)^50^ or the phosphorylation of WD-repeat protein interacting with phosphoinositide 2 (WIPI2), a positive regulator of autophagosome formation prone to proteasomal degradation upon mTORC1 phosphorylation^51^. Furthermore, mTORC1 is a repressor of the UPS. Upon rapamycin inhibition, the ubiquitination and subsequent proteasome degradation of long-lived proteins increases^52^. However, the biochemical mechanism by which mTORC1 suppresses UPS has not been identified and requires further studies.

#### 2.1.4. Lipid and nucleotide synthesis, mitochondrial metabolism, and biogenesis

Lipid and cholesterol synthesis is promoted by mTORC1 *via* sterol regulatory element-binding protein (SREBP)^53^^,^^54^ and peroxisome proliferator-activated receptor-*γ* (PPAR-*γ*)^55^. These transcription factors regulate the protein expression of lipid and cholesterol homeostasis-related genes. SREBP activation is promoted by low sterol levels in the cell or by the mTORC1-S6K1 pathway^56^. In addition, mTORC1 phosphorylates the SREBP suppressor lipin-1, which prevents its nuclear entry and attenuates SREBP inhibition^57^.

The mitochondrial oxidative function is balanced by mTORC1, as its inhibition by rapamycin decreases the activity of the peroxisome-proliferator-activated receptor coactivator (PGC)-1*α* and yin-yang1 (YY1) transcriptional complex^58^. Furthermore, Schieke et al. observed decreases in mitochondrial membrane potential, reductions in oxygen consumption and cellular ATP levels, as well as modified mitochondrial phosphoproteomes as a result of mTORC1 inhibition^59^.

Lastly, mTORC1 intervenes in *de novo* pyrimidine synthesis by the S6K1-dependent activation of carbamoyl-phosphate synthetase (CAD)^60^ and in purine synthesis by increasing the expression of MTHFD2 (mitochondrial tetrahydrofolate cycle component)^61^.

#### 2.1.5. Glucose metabolism

mTOR influences the glycolytic pathway at several points by the regulation of transcription factors, such as hypoxia-inducible factor 1-alpha (HIF1*α*) or Myc proto-oncogene protein (Myc)^62–64^. The effects of mTORC1 are mediated by its ability to promote the transcription of HIF1*α* while inhibiting glycogen synthase kinase-3 (GSK3). Inhibited GSK3 represses the phosphorylation of forkhead box protein k1 (Foxk1) and subsequently results in the accumulation of hypophosphorylated Foxk1, which induces HIF1*α* transcription^65^. HIF1*α* expression is also regulated at the level of translation, involving the mTORC1 target 4EBP1. Therefore, levels of genes encoding several glycolytic enzymes are indirectly modulated by mTORC1 *via* HIF1*α*. Another transcription factor influenced by mTORC1 is Myc, which stimulates genes involved in metabolism; however, its regulation by mTORC1 remains uncharacterised^64^^,^^66^.

### 2.2. mTORC1—upstream regulators

**Figure 3.**
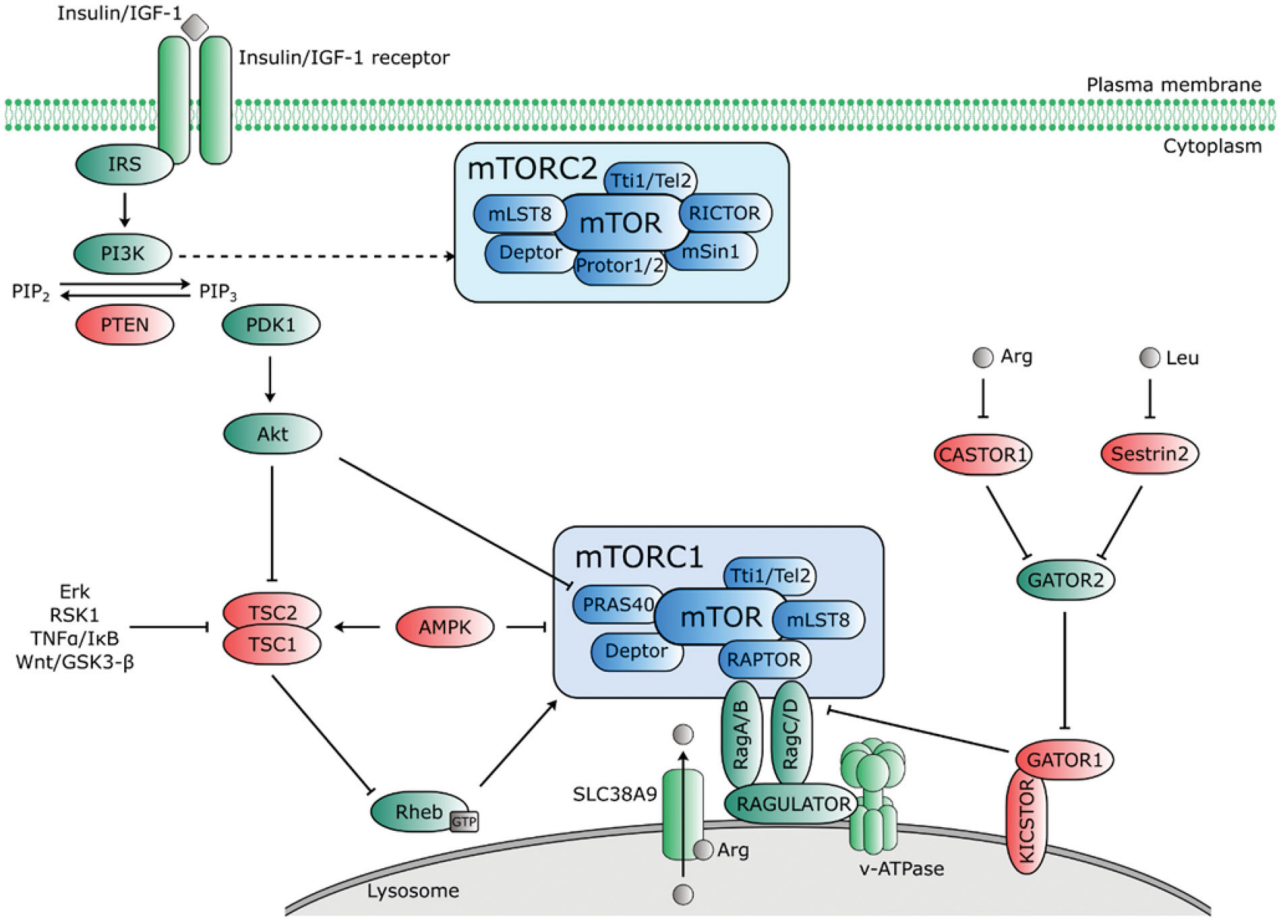
Upstream regulators of mTORC1 and mTORC2. Growth factors (insulin/IGF-1) bind to their receptors that phosphorylate IRS and activate PI3K. PI3K-IRS complex converts phosphatidylinositol-4,5-bisphosphate (PIP_2_) to phosphatidylinositol-3,4,5-trisphosphate (PIP_3_), which recruits phosphoinositide-dependent kinase 1 (PDK1) to activate Akt. PIP_2_-PIP_3_ conversion is counteracted by PTEN. Akt inhibits the TSC complex that acts as GTPase-activating (GAP) protein for Rheb. mTORC1 is activated by GTP-bound Rheb protein. Thus, when Akt activity is stimulated by PIP_3_, it phosphorylates TSC1/2 and switches off its inhibiting activity towards Rheb. Akt activates mTORC1 directly *via* phosphorylation of PRAS40. Amino acids induce activation of Rag proteins, which mediates translocation of mTORC1 to lysosomal surface. The intra lysosomal amino acids activate mTORC1 in an arginine-dependent manner *via* interaction of transporter SLC38A9 with the Rag-Ragulator-v-ATPase complex. Cytosolic amino acids engage the negative regulator GATOR1 (tethered to the lysosome by KICSTOR) and GATOR2. A direct leucine sensor Sestrin2 and arginine sensor CASTOR1 dissociate from GATOR2 in the presence of amino acids and release its inhibitory effect on GATOR1, thus positively regulate the mTORC1 pathway. AMPK negatively regulates mTORC1 either directly by Raptor phosphorylation, or *via* TSC1/2-Rheb axis. mTORC2 activation is PI3K-sensitive. Regulators of the mTOR pathway are depicted in green (positive) and red (negative). Dashed lines indicate indirect regulation.

Growth factors, such as insulin or insulin-like growth factor-1 (IGF-1), nutrients and amino acids, ATP, and stress regulate mTORC1 by different mechanisms, described in detail in Figure 3. Growth factors increase the activity of mTORC1 *via* PI3K-Akt^67–69^ and Ras^70^ signalling pathways.mTORC1 amino acid-sensitive activation is independent of tuberous sclerosis complex (TSC1/2). The activation pathway relies on the interaction between four Rag proteins (Ras-related GTP-binding proteins; GTPases) and Raptor in the presence of amino acids (especially leucine and arginine) and the subsequent localisation of mTORC1 to the lysosomal surface containing its known activator Rheb (Ras homolog enriched in brain)^71^. mTORC1 senses amino acids by two mechanisms (Figure 3). Intra-lysosomal amino acids require the transporter SLC38A9 to interact with the Rag-Regulator-v-ATPase complex for the arginine-dependent activation of mTORC1^72^^,^^73^, while cytosolic amino acids activate GATOR1 and GATOR2 complexes in mTORC1 activation. GATOR1, tethered to the lysosomal surface by the KICSTOR complex^74^, inhibits mTORC1 signalling, while GATOR2 acts as a positive regulator that interacts with GATOR1 at the lysosomal surface. In the presence of amino acids, the direct leucine sensor Sestrin2 and cytosolic arginine sensor CASTOR1 dissociate from GATOR2 and subsequently activate mTORC1^75–78^.

The processes controlled by mTORC1, such as the increase in cell mass, are energy-intensive. Under low cellular energy, AMP-activated protein kinase (AMPK) inhibits the mTORC1-dependent phosphorylation of S6K1 and 4EBP1 *via* the TSC1/2-Rheb axis, thus maintaining the energy balance of the cell. Additionally, AMPK reduces mTORC1 activity directly by Raptor phosphorylation^79^^,^^80^.

Induced expression of TSC2 and phosphatase and tensin homolog (PTEN) by DNA damage as well as AMPK activation by the induction of Sestrin 1/2 lead to decreased mTORC1 activity^81–83^. In addition to DNA damage, hypoxia inhibits mTORC1 signalling and upregulates the inhibitory protein REDD1/2 (DNA damage-inducible transcript 4 protein), which activates TSC1/2^84^. The mTORC1 pathway is coordinated by an S6K1-dependent negative feedback loop, which inhibits the PI3K-Akt axis upstream of PI3K; this pathway has profound implications for the treatment of tumorigenesis and metabolic diseases and the side effects of mTOR-based therapies (reviewed in Harrington et al. and Manning^85^^,^^86^).

### 2.3. mTORC2—structure, function, and regulation

**Figure 4.**
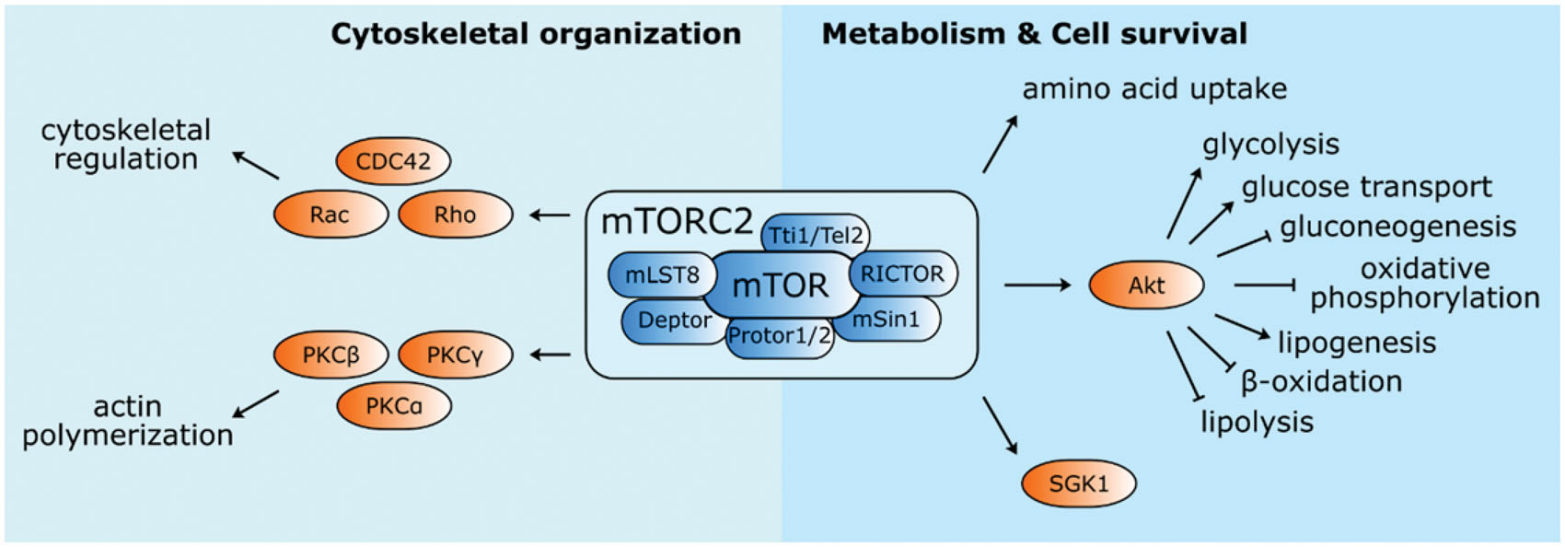
Structure and functions of mTOR complex 2. mTORC2 mainly phosphorylate several kinases from AGC family (PKC*α*, PKC*β*, PKC*γ*, PKC*ε*, PKC*η*/*Λ*, PKC*δ*, PKC*θ*, and SGK1). It is involved in the cytoskeletal organisation by modulating actin polymerisation and cytoskeletal structure regulation *via* PKCα and Rho family of small GTPases (Rac, Rho, and CDC42), respectively. mTORC2 also activates Akt, an important enhancer and suppressor of several metabolic pathways engaging mTORC2 in metabolism regulation and cell survival.

Our understanding of mTORC1 activity is mainly based on experiments involving the rapamycin-induced inhibition of its signalling pathway. However, mTORC2 shows acute resistance to rapamycin and our understanding of its function is therefore rather limited. mTOR complex 2 contains the mTOR protein interacting with Rictor^87^, Protor 1 or 2, mammalian stress-activated protein kinase interacting protein 1 (mSin1), mLST8 The Role of mTOR in Age-related Diseases, Tti1/Tel2, and Deptor^88^ (Figure 4). The two latter components exert inhibitory effects, identical to the mTORC1 complex. Rictor stabilises mTORC2 assembly and activity^89^, while mSin1 is involved in substrate recruitment and selection^90^. mLST8 is essential for mTORC2 function since its deletion completely suppresses complex activity. In contrast, mLST8 deletion has no clear effect on mTORC1^91^. The last complex component Protor 1 is required for serum/glucocorticoid-regulated kinase 1 (SGK1) activation *via* mTORC2^92^.mTORC2 mainly functions in the phosphorylation of several members of the AGC kinase family (e.g. PKC*α*, PKC*β*, PKC*γ*, PKC*ε*, PKC*η*/*Λ*, PKC*δ*, PKC*θ*, and SGK1)^93^^,^^94^. It regulates cell morphology by modulating actin polymerisation *via* protein kinase C*α*^95^. mTORC2 alters the cytoskeletal structure *via* the Rho family of small GTPases, including Rac, Rho, and Cdc42, and activates Akt, which indirectly stimulates mTORC1 activity and other effectors in the PI3K-Akt axis^89^.

Recent studies have indicated that the localisation of mTORC2 near the plasma membrane is important for its activation. The activity of plasma membrane-associated mTORC2 is continuous and autonomous of PI3K signalling, different from mTORC1. However, the activity of endosomal mTORC2 is PI3K-sensitive, suggesting the existence of several mTORC2 subpopulations^96^. Upstream, mTORC2 activity is controlled by the ubiquitination of mLST8 and Deptor^97^^,^^98^ and the phosphorylation or acylation of Rictor on several residues^99^^,^^100^. Additionally, ribosomes^101^, small GTPases^102^, TSC complex^103^^,^^104^, and amino acids^105^ contribute to mTORC2 regulation. The mechanisms underlying these regulatory interactions as well as the sensitivity of mTORC2 to other mTORC1 upstream regulators demand further studies.mTORC2 dysregulation is related to several human diseases, such as cancer, type 2 diabetes mellitus (T2DM), and ageing. As mentioned above, mTORC2 activates Akt, an important enhancer of aerobic glycolysis (*via* the regulation of glycolytic enzymes, such as hexokinase or 6-phosphofructo-2-kinase) and inducer of GLUT1 expression^106–108^. Moreover, Akt inhibits the activity of pyruvate dehydrogenase (PDH), a mitochondrial respiration enzyme^109^. mTORC2 upregulates glycolysis by inhibiting the phosphorylation of Class IIa histone deacetylases, leading to increased acetylation of forkhead box protein O1 (FoxO1) and FoxO3 and the subsequent upregulation of Myc, which activates various genes, such as lactate dehydrogenase (*LDH*) and the pyruvate kinase *PKM2*^110–112^. The regulation of glucose metabolism by increasing glucose uptake and glycolysis and inhibiting gluconeogenesis and oxidative phosphorylation might explain the abnormal glucose metabolism in T2DM and might contribute to the Warburg effect in cancer cells^113^.

In addition, there is compelling evidence that mTORC2 has a role in lipid metabolism; in particular, it increases lipogenesis and suppresses lipolysis and fatty acid *β*-oxidation *via* Akt and SREBP pathways^114^. mTORC2 dysregulation is linked to insulin resistance and lipogenesis-induced hepatocellular carcinoma *via* altered lipid metabolism^115–120^.mTORC2 also regulates amino acid uptake by increasing the expression of surface amino acid transporters^121^ and altering their activity by phosphorylation^122^. Direct mTORC2 phosphorylation of glutamate/cystine antiporter (xCT) decreases cystine uptake, thereby reducing the synthesis of glutathione and subsequently decreasing the cellular reactive oxygen species (ROS) buffering capacity^122^. The inhibition of mTORC2 by Torin 1 and Rictor silencing by siRNAs decrease nucleotide synthesis^123^. The mTORC2-mediated activation of Akt regulates nucleotide synthesis by modulating phosphoribosylpyrophosphate (PRPP) production and by altering the activity of the bifunctional purine biosynthesis protein ATIC^124^.

The exact role of mTORC2 in ageing remains unknown. Longevity studies using murine models have yielded conflicting results. In mouse models with depleted or deleted Rictor, lifespan is reduced in males but not in females, and the deleterious effect was independent of glucose intolerance^125^. On the other hand, in heterozygous Akt1 mice, the lifespan was increased^126^, suggesting that the role of mTORC2 in ageing and age-related diseases is complex and influenced by several factors.

## 3. Age-related diseases and mTOR

Several disorders have age as a risk factor, including neurodegenerative diseases (e.g. Parkinson’s disease [PD] and Alzheimer’s disease [AD]), cancer, and T2DM. The hyperfunction of mTOR has been observed in some of these disorders. Furthermore, the inhibition with rapamycin results attenuates the pathological processes in these diseases. However, there are still unresolved issues, including the extent to which mTOR regulates ageing *per se*, the extent to which mTOR influences pathologies, and the effectiveness of mTOR inhibition for the treatment of these diseases and even for slowing ageing. Although there is no simple answer, the amelioration of age-related diseases by mTOR inhibition is still a satisfying result of rapamycin therapy, because this approach may extend the lifespan, delay these pathologies, and promote healthy ageing.

In the text below, we provide a short description of the effects of mTOR and its inhibition on age-related diseases.

### 3.1. Type 2 diabetes mellitus

T2DM is an epidemic endocrine disorder with multifactorial causes. The prediabetic stage is characterised by insulin resistance and hyperinsulinemia, involving overworked *β*-islets. Progression to the second stage is associated with hypoinsulinemia and hyperglycaemia due to the failure of *β*-cell compensation for insulin resistance. Moreover, hyperamylinemia is found in insulin-resistant patients. The oligomerisation of amylin has a toxic effect on pancreatic *β*-cells, in which misfolded proteins alter endoplasmic reticulum (ER) homeostasis and cause prolonged unfolded protein response (UPR), resulting in *β*-cell death^127^.

In contrast to its general role in organisms, autophagy increases pancreatic *β*-cell survival. Ebato et al. and Jung et al. used mouse models with the deletion of Atg-7 (autophagy-related 7) in pancreatic *β*-cells^128^^,^^129^ and found that autophagy is necessary to maintain the structural and functional capacity of pancreatic *β*-cells in T2DM. Furthermore, autophagy protects against ER stress in insulin secretion-deficient *β*-cells and avoids the toxicity of human amylin (*h*IAPP)^127^^,^^130^.

The upstream regulation of autophagy by mTORC1 may be important in the progression of T2DM. In fact, an increase in pancreatic *β*-cell death and deterioration of autophagy has been observed in a mouse model of chronic mTORC1 overactivation. *β*TSC2^−/−^ mice show an early increase in *β*-cell mass and elevated insulin levels; however, the opposite effects are observed in older mice, showing a drop in *β*-cell mass and lower insulin levels. Older mice also accumulate ER stress markers, sequestosome-1 (p62/SQSTM1), and apoptosis markers^131^. In addition, an increase in mitochondrial mass and impaired mitophagy (mitochondrial autophagy) further support the link between autophagy impairment as a result of mTORC1 hyperactivation and mitochondrial dysfunction contributing to pancreatic *β*-cell failure^132^^,^^133^.

### 3.2. Alzheimer’s disease

AD is an age-related metabolic neurodegenerative disorder whose exact cause and pathogenesis remain unclear. It is defined by a progressive cognitive decline and formation of senile plaques and neurofibrillary tangles. Several theories for the aetiology of the disease include the *β*-amyloid (A*β*) cascade hypothesis^134–136^ and tau hyperphosphorylation hypothesis^137–139^. However, effective therapies based on these two theories are still lacking. Indeed, growing evidence indicates that dysfunctional cerebral glucose metabolism is a pathophysiological feature in AD^140–142^. Glucose transportation (depending on the function of astrocytes and glucose transporters)^143^ and intracellular oxidative catabolism (including glycolysis, the pentose phosphate pathway in the cytoplasm, Krebs cycle, and oxidative phosphorylation) are two main processes involved in cerebral glucose metabolism. In patients with AD, glucose transportation abnormalities and intracellular metabolic alterations occur due to insulin resistance and mitochondrial dysfunction, respectively^144^^,^^145^.

mTOR is highly expressed in the brain. mTORC1 and mTORC2 are fundamental for normal neuronal development (although the mechanism is still not fully understood) as well as for the maintenance of synaptic plasticity^146^. In the aged brain, mTOR is involved in translation and autophagy regulation, preventing the accumulation of toxic protein aggregates and neuronal degeneration (reviewed in Takei et al.^147^).

Analyses of both AD brains and mouse models have shown that mTOR signalling is upregulated during neurodegenerative development, with particularly high levels of 4EBP1 and S6K1 in the hippocampus and other areas^148^. Caccamo et al. detected mTOR hyperactivity in cell lines transfected with mutant amyloid precursor protein (APP) and in an animal model of AD (3 × Tg-AD mice), in which A*β*-induced mTOR hyperactivity is mediated by PRAS40^149^. Additionally, the continuous activation of the neuronal PI3K/Akt/mTOR axis in the AD brain causes insulin receptor substrate 1 (IRS1) inhibition, disabling normal insulin activation of this axis, providing a link between the pathology of AD and insulin resistance^150^^,^^151^.

Autophagy is altered in the AD brain and animal models of AD. The activity of this well-characterised effector pathway downstream of mTOR is reduced in AD due to the hyperactivation of the PI3K/Akt/mTOR axis, resulting in the reduced clearance and accumulation of protein aggregates. A*β* monomers and oligomers increase the activity of the PI3K/Akt axis, leading to autophagy inhibition as well as insulin receptor (IR) internalisation and inactivation, resulting in a vicious cycle^152^. Moreover, studies of mouse models and post-mortem analyses of AD brains suggest that there is an association between mTOR signalling and tau neuropathology, as evidenced by the observation that a dysfunctional autophagy-lysosome system promotes the formation of tau aggregates^153^^,^^154^.

In fact, the inhibition of mTOR by rapamycin eradicates cognitive deficits and reduces levels of A*β* in a transgenic mouse model of AD, in which autophagy is strongly activated in the hippocampus^155^. Several other studies have suggested that mTOR inhibition has positive effects on A*β* and tau levels in AD models. Cassano et al. found that everolimus-induced mTOR inhibition improves cognitive function and reduces APP/A*β* and tau levels in depressive-like phenotype in 3xTg-AD mice^156^. Tramutola et al. suggested that rapamycin has a neuroprotective effect on the hippocampus in Ts65Dn (an established mouse model of Down syndrome); in particular, mTOR inhibition led to autophagy and insulin signalling recovery, reductions of APP and tau hyperphosphorylation levels, and reductions in the levels of oxidative stress markers^157^.

### 3.3. Other neurodegenerative diseases

PD, the second most prevalent neurodegenerative disease, is characterised by the presence of Lewy bodies—aggregates composed of α-synuclein and poly-ubiquitinated proteins. The loss of dopamine neurons in the substantia nigra results in motor symptoms (muscular rigidity and tremor) as well as in non-motor somatic and psychological manifestations^158^. In addition to genetic mutations, ageing and dopaminergic neuron-specific toxins (e.g. 6-hydroxydopamine, rotenone, and 1-methyl-4-phenyl-1,2,3,6 tetrahydropyridine) are the main causes of disease progression^159^. mTOR signalling participates in several stages of PD in both active and inactive states. α-Synuclein accumulation has been observed in the cerebral cortex of patients with PD, who shows elevated levels of mTOR protein expression^160^. α-Synuclein aggregates eventually worsen disease progression by autophagy inhibition in response to increased mTOR activity. The rapamycin-induced inhibition of mTOR can restore the autophagy impairment, leading to the clearance of neurotoxic aggregates^161^^,^^162^.

Nonetheless, the attenuation of mTOR activation, either by Akt phosphorylation or AMPK activation, has been described in different cellular models of PD. Since the mTOR pathway is important for cell proliferation and survival, Akt or AMPK-dependent mTOR downregulation by PD toxins leads to the impairment of protein synthesis and neuronal cell death. The overexpression of functional mTOR can partially restore this condition^163^^,^^164^.

Considering the role of mTOR in the pathogenesis of PD, a balance between mTOR activation and inhibition should be maintained, as increased autophagy would ameliorate α-synuclein accumulation, while mTOR-regulated cellular functions (e.g. synaptic plasticity and memory formation) would remain intact.

Many neurodegenerative disorders are associated with pathological intra-neuronal protein aggregates. The importance of autophagy in neuronal health and the impact of autophagy dysfunction in neurodegeneration has been confirmed, thus emphasising the key role of mTOR^165^. For instance, the mutant form of the huntingtin protein (mHTT), responsible for the formation of pathogenic aggregates in Huntington’s disease, may mediate autophagy by different mechanisms. Both wild-type and mutant HTT interact with autophagy-associated proteins and influence the autophagy pathway, and both forms are degraded by autophagy^166^^,^^167^. Rapamycin-induced mTOR inhibition attenuates HTT toxicity in an animal model of HD and has a neuroprotective effect *via* autophagy activation^168^^,^^169^.

Moreover, there is a tight connection between SOD1 (superoxide dismutase; in which toxic gain-of-function mutations lead to aggregation in amyotrophic lateral sclerosis [ALS]) and autophagy dysfunction^170^. On the one hand, mutant SOD1 increases mTOR-dependent autophagy^171^^,^^172^; on the other hand, autophagy fails to degrade the products of mutant *SOD1*^173^^,^^174^. The autophagic degradation of mutant SOD1 is believed to be beneficial in ALS. However, rapamycin itself has shown both beneficial and harmful effects; for example, it promotes motor neuron degeneration and shortens the lifespan of mice^175^ but simultaneously increases the survival of ALS mice deficient in mature lymphocytes^176^. Owing to these contradictory results, the exact roles of mTOR-dependent autophagy and rapamycin in ASL require further research.

### 3.4. Cancer

During tumorigenesis, a high rate of proliferation demands an increased supply of nutrients and energy. Accordingly, one of the hallmarks of cancer cells is metabolic reprogramming, known as the Warburg effect^177^^,^^178^. A metabolic shift towards glycolysis and lactic acid fermentation (instead of oxidative metabolism) influences the tumour microenvironment and eventually suppresses antitumor immunity as well as the expression of cell surface markers^179^. mTOR alters the metabolism of glucose, lipids, amino acids, and nucleotides according to the immediate demands of a cell, and the hyperactivation of its pathway may confer an advantage to cancer cells.

Mutations in the PI3K/Akt/mTOR signalling pathway contribute to the Warburg effect by increasing the expression of glucose transporters and glycolytic enzymes in cancer cells *via* the HIF-1*α* and Myc pathways^110^^,^^180–182^. The PI3K/Akt/mTOR signalling axis also increases lipid metabolism in tumours by the production of lipogenic enzymes *via* SREBP activity^183^ and decreases antitumor immunity by reducing PGC1*α* expression in tumour-infiltrating lymphocytes, resulting in their exhaustion^184^. The phosphorylation of the mTORC1 effector S6K1 promotes pyrimidine and purine synthesis, which is required for massive DNA replication in cancer cells^60^^,^^61^. In addition, loss-of-function mutations in the tumour suppressor PTEN and mutations in the Ras signalling pathway might drive oncogenic changes in the mTOR pathway^185^.

Mutations that constitutively hyperactivate mTOR contribute to cancer^186^. Mutations have been reported in the mTOR protein and mTOR complex components, e.g. Rictor, in breast cancer and lung cancer^187^^,^^188^. The dysregulation of the mTORC1 downstream effectors 4EBP1/eIF4E promotes the translation of pro-oncogenic proteins involved in angiogenesis, cell survival, metabolism, and metastasis^189^.

The mTOR signalling pathway is the second most frequently altered pathway in human cancers (after the p53 pathway)^190^; accordingly, extensive research has focussed on the role of mTOR in cancer. Furthermore, the development and application of appropriate mTOR inhibitors or compounds targeting the dysregulated PI3K/Akt/mTOR axis are key areas of research. Rapalogs, ATP-competitive inhibitors, PI3K/mTOR dual inhibitors, and RapaLink have all been evaluated in pre-clinical and clinical studies of various types of tumours^191^. A detailed description of this topic is beyond the scope of this review; for a more comprehensive overview, see the reviews by Magaway et al.^191^ and Mossmann et al.^192^

## 4. mTOR in ageing

Ageing can be defined as functional decline causing age-related diseases and eventually death. Several theories regarding the mechanism underlying ageing have been proposed, among which the Hayflick limit and the ROS theory are the most widely known. The Hayflick limit is based on a 1965 study showing that fibroblasts have a limited replicative lifespan due to telomere shortening during each replication^193^. However, this theory is not universally supported, since a clear correlation between telomere length and the maximal lifespan has not been observed^194–196^. The ROS theory was proposed by Harman in 1956^197^^,^^198^, who stated that “there had to be some common, some basic cause which is killing everything. Free radicals cause random damage, and depending on the type of radical, they can cause all kinds of damage from day one.” However, clinical trials have disproven this theory, since antioxidants have no effect on age-related diseases or mortality and do not extend lifespan. Indeed, lifespan can be extended without reductions in ROS^199^^,^^200^.

Blagosklonny proposed an alternative theory in which cell senescence is considered a quasi-program of post-development. Development is a highly regulated program, and senescence is a continuance of this program and is constantly switched on, eventually becoming hyper-functional and damaging. Ageing and age-related diseases are, therefore, initially described as cellular hyperfunctions (apoptosis resistance, hypertrophy, and large cell morphology), involving increased growth and increased nutrient intake, and secondly as a loss of function. Age-related diseases, such as hypertension, cancer, or atherosclerosis, result from hyperfunction and hypertrophy (e.g. atherosclerosis involves smooth muscle cell, macrophage, and platelet hyperfunction)^201^. Cell senescence might be induced by different stimuli that positively regulate the CDK inhibitors p21 and p16^202–204^. Such stimuli include oxidative stress, microtubule-stabilising agents, retinoids, DNA-damaging agents, oncogenes, and mitogenic signalling *via* mitogen-activated protein kinase (MAPK) cascades that block the cell cycle but do not block cell growth, resulting in a hypertrophic phenotype typical of senescent cells^205–211^.

López-Otín et al. proposed nine tentative hallmarks of ageing, including telomere attrition, epigenetic alterations, genomic instability, loss of proteostasis, deregulated nutrient sensing, mitochondrial dysfunction, cellular senescence, stem cell exhaustion, and altered intercellular communication; these appear to be common features of ageing across taxa^212^. Although there are obvious links between hallmarks of ageing and previously described theories, telomere attrition or genomic instability alone are not a cause of ageing but rather a component of a complex mechanism.

Some of the mentioned hallmarks, namely a loss of proteostasis, deregulated nutrient sensing, mitochondrial dysfunction, cellular senescence, and stem cell exhaustion, are affected by mTOR hyperactivation or dysregulation. Hence, as a key factor in the process of ageing, the roles of mTOR in these hallmarks are briefly described below.

### 4.1. Loss of proteostasis

The term proteostasis describes several cellular processes that maintain the appropriate proteome within the cell. Proteostasis includes the initial synthesis of nascent proteins, correct folding, transport and secretion of mature proteins, and degradation of damaged proteins. A decrease in proteostasis is related to ageing and some age-associated pathologies (e.g. AD)^213^. By the regulation of cap-dependent and cap-independent mRNA translation, mTORC1 can potentiate the synthesis of translation/ribosomal-related proteins. mTORC1 inhibition, leading to a general decrease in proteosynthesis, might slow down ageing by reducing oxidative and proteotoxic stress (however, direct evidence for this effect has not been obtained)^214^. In fact, protein quality control and translation are interconnected *via* mTOR, whose activity is also regulated by chaperone availability^215^. Moreover, lifespan might be prolonged by the deletion of the translational regulator S6K1^216^. In addition to being an important regulator of protein synthesis, the function of mTOR as an autophagy regulator has a crucial role in the maintenance of proteostasis (along with the UPS). Thus, the inhibition of mTORC1 inhibits the age-related decline in autophagy and loss of proteostasis^217^.

### 4.2. Deregulated nutrient sensing

The insulin/PI3K/Akt signalling pathway upstream of mTOR responds to growth factors and nutrients and is deregulated in ageing organisms. Increased mTOR activity is linked to insulin resistance, commonly observed in ageing organisms (negative feedback with insulin/PI3K/Akt signalling) and age-related obesity (evidenced by the increased activity of mTOR in hypothalamic neurons in ageing mice)^218^^,^^219^. Therefore, it is unsurprising that a caloric restriction decreases mTORC1 activity and promotes lifespan and health in all investigated eukaryotes to date^220–222^.

### 4.3. Cellular senescence

mTOR, as a central regulator of nutrient conversion into biomass, plays an important role in cell senescence and the hypertrophic phenotype. Its activity is associated with ageing as well as senescence-associated diseases (metabolic syndrome, hereditary tumours, osteoporosis, AD, and macular degeneration). The loss of proteostasis, accumulation of mitochondrial and lysosomal mass, shifts in metabolism, and failure of autophagy are all associated with senescent cells and constitutive mTORC1 signalling^223^. Moreover, the senescence-associated secretory phenotype (SASP) contributes to ageing by the secretion of proinflammatory mediators and is promoted by mTORC1^224^^,^^225^. Senescent cells steadily accumulate with age as a result of proinflammatory and pro-oxidant signal production, inducing inflammation and suppressing apoptosis. In fact, the inhibition of mTORC1 activity, as well as the activity of its effectors, results in SASP inhibition, lifespan extension, and the amelioration of several age-related diseases^226^.

### 4.4. Mitochondrial dysfunction

As mentioned above, mTOR is involved in the control of cellular energy metabolism by regulating mitochondrial functions and mitochondrial biogenesis. An increase in mitochondria is associated with ageing and age-related diseases^227^. In fact, mutations activating mTOR signalling increase the mitochondrial DNA number copy as well as oxidative metabolism-related gene expression, suggesting that the ageing-associated hyperactivation of mTOR also increases the number of mitochondria and alters mitochondrial function (*via* PGC-1 and YY1)^58^^,^^228^. Additionally, mTORC1 inhibition not only attenuates these increases in mitochondria but also stimulates autophagy (in particular, mitophagy), thereby clearing old and dysfunctional mitochondria. Thus, preserved number and function of mitochondria *via* mTOR inhibition might slow down ageing and alleviate age-related diseases^227^^,^^229^.

### 4.5. Stem cell exhaustion

Age-related dysfunctions in tissues might be caused by declines in the amount and function of stem cells, thus lowering the tissue regenerative potential. The upstream hyperactivation of mTOR due to the deletion of PTEN or TSC1 or the constitutive activation of Akt lessens the number and function of haematopoietic stem cells (HSCs)^230–232^. Rapamycin treatment restores the self-renewal capacity of mouse HSCs^233^, enhances intestinal stem cell function in Paneth cells^234^, and generally promotes stem cell function by reprogramming somatic cells to generate induced pluripotent stem cells^235^. Taken together, mTOR inhibition might reverse the ageing phenotype by inducing stem cell rejuvenation, and this might expand lifespan and restore pathophysiological changes in some age-related diseases.

## 5. Expert opinion

The discovery of a pharmacological approach to slow ageing is considered the Holy Grail of medicine. The finding that mTOR inhibition prolongs lifespan and postpones the onset of age-associated diseases in mammals prompted substantial interest in the development of mTOR inhibitors as drugs to augment human longevity. However, interventions targeting the mTOR signalling pathway, a central switch in cellular metabolism, might be both a valuable and dangerous strategy. Since the pharmacological inhibition of mTOR by rapamycin and rapalogs is an FDA-approved clinical principle, there is substantial information about side effects. The most common side effects are immunosuppression, hyperglycaemia^236^, dyslipidemia^237^, as well as interstitial pneumonitis^238^. Most of these side effects are dosage-dependent and may regress with lower dosages. Moreover, rapalogs are limited by the lack of tissue specificity and unwanted disruption of mTORC2. Thus, further efforts should focus on the development of mTOR-targeting therapeutics outside of these two modalities, such as truly mTORC1-specific inhibitors for use in diabetes, neurodegeneration, and life-span extension or tissue-specific mTORC1 agonists for use in muscle wasting diseases and immunotherapy. It is also worth noting that rapamycin and rapalogs have mostly been tested in combination with other drugs, such as steroids or proliferation inhibitors, in patients with severe disease; preventive mTOR inhibitor therapy in the general healthy population might be more tolerable. Considering that the mTOR signalling pathway is involved in multiple biological processes, it is an attractive candidate for research. We should learn how to take advantage of its beneficial effects alone while minimising side effects. Additionally, the discovery of such specific mTORC1 therapeutics might shed light on key biological questions regarding the link between metabolism, immune responses, and ageing.

## 6. Conclusions

mTOR is a key factor in several hallmarks of ageing. Its signalling pathway directly or indirectly affects common determinants of ageing in various taxa. However, our understanding of ageing and the underlying mechanisms is still limited. Most experiments are based on the inhibition of mTOR or its effectors either genetically or by rapamycin. Future studies of ageing and age-related disease face several challenges. First, the mechanism underlying lifespan extension has not been characterised in a broad range of taxa. Theories for the roles of mTOR, though persuasive, require further investigation. Second, the life-prolonging effect of mTOR inhibition by rapamycin in mammals might not be directly applicable to humans. Rapamycin is an FDA-approved immunosuppressant; however, its chronic use results in several side effects (e.g. hyperglycaemia, dyslipidemia, and interstitial pneumonitis). Moreover, the mTOR signalling pathway is highly complex, and its inhibition might result in the undesirable activation or inhibition of other related pathways, leading to the development or worsening of pathologies (e.g. insulin resistance or tumorigenesis). Available mTOR inhibitors tested on animal models or in clinical trials do not show sufficient effects on lifespan or age-related disorders as a result of their physicochemical properties or side-effects.

The enigma of ageing is still unresolved, and successful lifespan-extending interventions represent a challenging task for scientists, with important implications in the context of the expanding social-economic problem that ageing is becoming. It is important to set reasonable goals aimed at (i) a better understanding of the mTOR signalling pathway; (ii) the discovery of effective inhibitors with appropriate properties; and (iii) the development of interventions that would not only add years to the lifespan but would also support healthy ageing and delay age-related disorders.
